# Effect of Rebound Exercises and Circuit Training on Complications Associated with Type 2 Diabetes: Protocol for a Randomized Controlled Trial

**DOI:** 10.2196/resprot.8827

**Published:** 2018-05-07

**Authors:** Bashir Kaka, Sonill Sooknunan Maharaj

**Affiliations:** ^1^ Discipline of Physiotherapy School of Health Sciences, College of Health Sciences University of KwaZulu-Natal Durban South Africa

**Keywords:** diabetes, musculoskeletal pain, exercise

## Abstract

**Background:**

The incidence of type 2 diabetes mellitus, a chronic lifestyle disease, and its complications are on the rise. Exercise has been documented as being effective in the management of musculoskeletal pain, depression, and reduction of hyperglycemia in diabetic patients. However, there is no consensus regarding the types of exercise that reduce musculoskeletal pain and depression and improve quality of life as well as respiratory function among individuals with type 2 diabetes.

**Objective:**

The objective of this study is to determine the effects of rebound and circuit training on musculoskeletal pain, blood glucose level, cholesterol level, quality of life, depression, and respiratory parameters in patients with type 2 diabetes mellitus.

**Methods:**

A total of 70 participants are expected to be recruited in this single blind randomized controlled trial. Computer-generated random numbers will be used to randomize the participants into 3 groups, namely, the rebound exercise group, the circuit exercise group, and the control group. Measurements will be taken at baseline and at the end of the 8 weeks of the study. Participants’ musculoskeletal pain will be assessed using the visual analog scale, quality of life will be assessed using the SF 12 Health Survey Questionnaire, depression using the Beck Depression Inventory, respiratory parameters using the spirometer, and biochemical parameters such as glucose level and cholesterol level using the glucometer. Data will be analyzed using descriptive statistics and inferential statistics of multivariate analysis of variance between the groups and paired t test within the group. Alpha will be set at .05.

**Results:**

The results of this study will identify the effectiveness of rebound exercise and circuit training, compared with the control, in the management of type 2 diabetes mellitus and on quality of life, musculoskeletal pain, depression, glycemic control, cholesterol level, as well as improvement in respiratory function.

**Conclusions:**

Though different additional strategies such as exercise and dietary and lifestyle modifications exist for the control of type 2 diabetes, they are mostly applied for the control of glucose level. No strategies have been identified for the control of complications associated with diabetes such as musculoskeletal pain, depression, and reduction in quality of life.

**Trial Registration:**

Clinicaltrials.gov NCT03200795; https://clinicaltrials.gov/ct2/show/NCT03200795 (Archived by WebCite at http://www.webcitation.org/6mBgcj6z7)

## Introduction

### Overview

Diabetes is a global epidemic disease [1]. The global prevalence of diabetics is currently estimated to be 415 million and is projected to rise to over 642 million by the year 2040, with Asians suffering the bulk of the total diabetes epidemic [2]. The International Diabetes Federation (IDF) estimated that there were 14.2 million diabetes patients living in Africa in 2015, excluding the 67% of patients that are undiagnosed, and the projection rate is expected to rise to 34.2 million by 2040 [2]. South Africa is among the most populous African countries with the highest number of people with diabetes with 2.3 million people, followed by Democratic Republic of Congo 1.8 million, Nigeria 1.6 million, and Ethiopia 1.3 million. Nearly half of the adults with diabetes live in these countries. In South Africa, the prevalence of diabetes mellitus (DM) is estimated to be about 6%, excluding the undiagnosed cases, whereas 9% of the population has prediabetes. DM accounts for 58 deaths daily and is the fifth highest cause of natural deaths [3]. The highest prevalence of diabetes is among the Indian population (11%-13%), as this group has a strong genetic predisposition to diabetes. This is followed by 8% to 10% in the colored community, 5% to 8% among blacks, and 4% among whites. The prevalence of diabetes in the public health sector of KwaZulu-Natal was found to be 14.3% higher than the national prevalence estimates [3].

Diabetes is a systemic disease that affects multiple body functions and structure. The typical spectrum of problems of patients with DM can now be defined based on the International Classification of Functioning, Disability and Health (ICF). For practical purposes and in line with the concept of condition-specific health status measures, DM problems can be classified as follows: body function as hematological system function, sensation of pain, musculoskeletal system, and respiratory system. On the basis of activities and participation restriction, DM problems can be classified as quality of life (QoL) and psychosocial factors. These will serve as a guide in clinical studies with DM or as a guide to multidisciplinary assessments in patients with DM [4].

In 2004, the US National Health Interview Survey reported that 58% of diabetic patients would have a functional disability [5]. The percentage of diabetic patients with a functional disability will increase as the number of diabetic patients increases and will constitute a major public health problem. Recent data show that the prevalence of musculoskeletal pain (MSKP) manifestations in the hands and shoulders in patients with type 1 or type 2 diabetes is 30%. These manifestations are closely linked to age [6] and prolonged disease duration [7,8].

Musculoskeletal disorders are very common in diabetes and are associated with poor glycemic control and more complications. Assessment of musculoskeletal disorders among diabetics should include an estimate of cholesterol, glycemic control, MSKP, respiratory parameters, and QoL.

People with diabetes are at twice as high a risk of suffering from premorbid depression as the general population [5]. The coexistence of depression in people with diabetes catalyzes serious disease comorbidities, MSKP, decreased respiratory capacity, poor glycemic control which may lead to hyperlipidemia and poor QoL, and escalated health care expenditures [9]. Depressive symptoms are more likely to persist among persons with multiple diabetic-related complications such as musculoskeletal disorders. Increase in pain may lead to increased depression and reduction in respiratory capacity and QoL. Studies have shown that both exercises and pharmacotherapy [10] can decrease depression and improve glycemic control and overall QoL in persons with diabetes and thus, in addition, provide substantial financial savings and improved medical care for these individuals.

Exercise, in addition to diet modification and medication, has long been recommended as one of the 3 main components of diabetic therapy [11]. The low cost and nonpharmacological nature of exercise enhances its therapeutic appeal among type 2 diabetic patients. Published exercise intervention trials [12-15], using different types of intervention, usually have small sample sizes and the findings of their studies have varied. The optimal type, frequency, intensity, and duration of exercise for achieving therapeutic goals in type 2 diabetes are not known. The psychosocial variables and cardiorespiratory-related health of diabetics appear to be underexplored in exercise-related interventions [11]. Moreover, prospective randomized controlled trials (RCTs) on the effects of exercise in the prevention or treatment of musculoskeletal complications and disability seen in diabetes patients are needed [16].

Exercise has been documented as effective in the management of MSKP; however, there is no consensus regarding the type of exercises that improve MSKP. The effectiveness of exercise in reducing MSKP as well as depression and in improving respiratory function among individuals with type 2 diabetes has not been documented.

Rebound exercise is a type of exercise that can aid the management of type 2 diabetes [17] and is likely to improve MSKP and depression. Circuit resistance training (CRT) has recently been documented to be safe in the management of type 2 diabetes; however, its effects on MSKP and depression have not been assessed [1]. Rebound exercise on the mini trampoline moves all parts of the body at once, so it is also called cellular exercise [18]. It may be superior to any other type of exercise as it utilizes gravity and forces of acceleration and deceleration so that at the top of the bounce one experiences weightlessness and at the bottom the weight doubles pulling into the center of the rebounder. Rebound exercise is also a form of aerobic exercise that increases oxygen consumption and stimulates immune response.

Circuit training is a combination of strength training and aerobic exercises. Strengthening exercise is a resistance exercise that helps to keep the muscles flexible and strong and also strengthens the bones. Aerobic exercise, on the other hand, is a less vigorous exercise that increases oxygen consumption; the increase in oxygen consumption helps to burn more calories and stimulates immune response and cardiovascular health [19]. Rehabilitation can assist to retain physical and functional abilities as well as control psychological emotions [20]. Therefore, comprehensive rehabilitation to manage diabetes-related complications that encompasses exercises such as rebound and circuit training may improve glycemic control, reduce depression, and improve QoL and musculoskeletal functions.

### Theoretical Framework

Previous studies have assessed the effect of combined resistance and aerobic exercises on type 2 DM patients and observed significant reductions in HbA_1c_ [21-23]. However, Geirsdottir et al [24] did not observe any favorable changes in fasting glucose or HbA_1c_ in patients with type 2 diabetes. Therefore, there is no consensus on the effect of CRT in type 2 diabetes. Rebound exercise has been documented to be effective in reducing plasma fasting glucose, HbA_1c_, and body mass index (BMI) in type 2 diabetes. It is also simple, inexpensive and enjoyable [23]. Moreover, it has been documented to improve QoL in patients with type 2 diabetes [17]. However, its effect on MSKP, respiratory parameters, and depression has not been documented. Prospective RCTs on the effects of exercise in the prevention or treatment of musculoskeletal complications, cardiorespiratory parameters, and psychosocial variables seen in diabetes patients are needed [16]. In African countries, there is a paucity of reports that describe musculoskeletal disorders in diabetic patients. To the best of our knowledge, no clinical trial has been conducted that compares the effect of rebound exercises and circuit training on MSKP, respiratory parameters, and psychosocial variables among patients with type 2 diabetes. No previous studies had been conducted to assess the effect of any treatment or prevalence of MSKP manifestations in diabetic patients or to evaluate the predisposing factors. This study is, therefore, proposed to determine the effect of rebound exercises and circuit training on MSKP, as well as selected biochemical and psychosocial factors among individuals with type 2 diabetes.

### Objective

The objective of this research is to investigate the effect of rebound exercises and circuit training and compare them to the routine care of type 2 diabetic patients. The objectives of this research are as follows;

1. To investigate the effect of rebound exercises on glycemic control, cholesterol level, respiratory parameters, pain scores, depression, and QoL among type 2 diabetes patients
2. To investigate the effect of CRT on glycemic control, cholesterol level, respiratory parameters, pain scores, depression, and QoL among type 2 diabetes patients
3. To investigate the effect of routine care (control group) on glycemic control, cholesterol level, respiratory parameters, pain scores, depression, and QoL among type 2 diabetes patients
4. To compare the effect of circuit training, rebound exercises, and routine care on glycemic control, cholesterol level, respiratory parameters, pain scores, depression, and QoL among type 2 diabetes patients.

## Methods

### Study Design

The study is a single-blind, parallel RCT. RCTs are quantitative, comparative, controlled experiments in which investigators study 2 or more interventions in a series of individuals who receive them in random order. The RCT is one of the simplest and most powerful tools in clinical research.

### Participants

The participants for the study will be patients diagnosed with type 2 diabetes by the attending physician at King Edward Hospital. Participants will be considered eligible if they have been diagnosed with type 2 diabetes for at least 4 years and have been on oral hypoglycemic control. They will be recruited consecutively as they are available through the outpatient endocrinology clinic of the hospital by a member of the research team. Only participants with MSKP and depression will be included in the study. They will be screened for MSKP using the Nordic Musculoskeletal Symptoms Questionnaire and for depression using the Beck Depression Inventory (BDI). The participants must be aged between 20 and 55 years. Those within this age limit are considered to be adults with musculoskeletal affectation, as indicated by Nordic Musculoskeletal Symptom Questionnaire, and with at least ≥11 score on the BDI. Only participants who meet the inclusion criteria will be considered for the randomization into the groups. The consent of the participants will be sought before they participate in the study.

Patients will be excluded from the study if they are involved in sporting activities or if their musculoskeletal problems are severe and may prevent them from performing some of the exercises. Moreover, individuals with hypertension, coronary artery disease, or myocardial infarction; or who have had cardiac or abdominal surgery within the previous 6 months; or who have a history of fractures of the spine, hip, knee, or ankle joints; as well as those with lower limb weakness and deformities with a loss of protective sensation in the feet will be excluded. Similarly, pregnancy or lactation, use of insulin, and the presence of retinopathy or nephropathy will make a patient ineligible. Finally, subjects who do not consent to participate will be excluded from the study. Participants who meet the inclusion criteria will be randomized into 1 of the 3 groups of the study.

### Setting

The research will be conducted at the Performance Laboratory of the Department of Physiotherapy, School of Health Sciences, University of KwaZulu-Natal. The participants will be recruited from the King Edward Hospital, Durban. King Edward Hospital is a tertiary hospital that provides specialist services to the people of KwaZulu-Natal. It is located in Durban City, Ethekwini Municipality, KwaZulu-Natal Province, South Africa.

### Study Team

The study team will consist of 4 physiotherapists, of which 2 are clinicians and the other 2 are both clinicians and researchers. The team will also include an endocrinologist who is a physician with 10 years of research experience. In addition, 2 among the physiotherapists will be research assistants and they will administer the treatment. The endocrinologist will screen the patients and ensure that they take the same medications and that they are medically fit, whereas the researchers will screen the participants to ensure that they meet the inclusion criteria.

### Intervention

The study will comprise 3 groups; the rebound exercise group, the circuit resistance exercise training group, and the routine care group (the control). The routine care group will undergo one exercise training after 8 weeks of enrollment. Participants will be monitored for subjective fatigue, dyspnea, respiratory distress, profuse sweating, or unsteady gait during the exercise activities, as these may be indications that they have reached maximum exertion.

### Rebound Exercise Group

Participants randomized to this group will be instructed on the proper techniques of the desired movements (hopping) on the rebounder. All instruction and training will be provided by the research assistants who are physiotherapists practicing in South Africa. They will teach the participants how to rebound during the session. To ensure exercise endurance, patients allocated to this group will complete a 6-min walk test (6MWT), as prescribed by the American Thoracic Society [25], which will be conducted along a straight 40-m Department of Physiotherapy passageway. On successful completion of the 6MWT, patients will be given an opportunity to familiarize themselves with the equipment. Each participant will undergo 3 sessions a week for 8 weeks, with each session lasting between 15 and 25 min.

The exercise will involve bouncing on the center portion of a mini trampoline (2013 Portable Model Half-Fold Cellerciser) with feet slightly apart and knees in full extension. Each foot strike equals one step or bounce with step height. This will be defined as the distance between the foot at a maximum height of the jump and the bed of the center of the trampoline ranging between 10 and 15 cm [23]. Exercise durations were set at 5-min intervals with 3-min rest periods. Each exercise program session will last 15 min with 9 min of rest for the first 4 weeks, progressing to 25 min with 15 min of rest in the subsequent 4 weeks, 3 times per week over 8 weeks.

Bouncing frequency will be determined by signals from a metronome and will be set at between 90 and 120 bounces per minute. Heart rate training zone will be maintained during the exercise at a moderate intensity of 40% to 60% and will be calculated using the Karvonen formula ([heart rate reserve×training percentage]+resting heart rate) [23]. The heart rate will be monitored using the heart rate monitor (Polar Electro Oy) that will display the participant’s heart rate and emit a warning signal if the heart rate goes outside the prescribed training zone, thus serving as a guide and indicator for the participant to adjust the steps and bounce while on the trampoline. In addition, the Borg scale will be used to assess the rate of perceived exertion.

### Circuit Exercise Group

The circuit exercise for the participants in this group will be designed for each participant. To ensure exercise endurance, patients allocated to this group will complete a 6MWT, as prescribed by the American Thoracic Society [25], which will be conducted along a straight 40-m Department of Physiotherapy passageway. On successful completion of the 6MWT, patients will be given an opportunity to familiarize themselves with the circuit exercise equipment. Exercise sessions will take place 3 times a week for 8 weeks. The participants will undergo 10 min of warm up before and 10 min of cool down after the training. The stations for circuit training include 2 stations—1 and 2. Station 1 is the resistance exercise, whereas station 2 is the aerobic exercise with the bicycle ergometer. Station 1 comprises the following 6 substations: station A—bench press, station B—seated row, station C—lateral pull down, station D—biceps forward, station E—front thigh and back thigh, and station F—leg press. Resistance exercises will be performed on weight machines. Throughout the resistance training program, participants will rotate among the different machines until they have used every one: the bench press, seated row, lateral pull down, biceps forward, front thigh, back thigh, leg press, and rowing.

Participants will be instructed to exhale while lifting a weight and inhale while lowering it, to minimize blood pressure excursions, and to rest for 2-3 min between sets. Participants will perform 1 set of resistance exercise 3 times weekly for the first 2 weeks, 4 sets of each resistance exercise 3 times weekly during weeks 3 and 4, and complete set of resistance exercise in the last 4 weeks. For each station, the participants will spend 10 min; this includes the substations. Resistance will be increased by 5-10 kg, once the participant is able to perform more than 15 repetitions while maintaining proper form.

All the aerobic activities of the circuit training will be performed on a cycle ergometer. Participants will be free to vary the machine used from one visit to the next. Exercise intensity will be standardized by using heart rate monitors (Polar Electro Oy) that will display the participant’s heart rate and emit a warning signal if the heart rate goes outside the prescribed training zone, thus guiding the participant to adjust the workload up or down to achieve the desired intensity. In addition, the rate of perceived exertion will be measured using the Borg scale.

### The Routine Care Group (Control Group)

Participants in the control group will receive routine medication as prescribed by the attending physicians at the diabetes clinic and will be monitored. Their prescriptions will be guided by the IDF guidelines. After 8 weeks of routine care, the participants will be enrolled into either the rebound exercise or circuit exercise group. The control group will be given health magazines to read, whereas the exercise groups will be engaged in the exercise programs.

All the 3 groups will receive routine medication and counseling regarding diet, weight control, and information to identify, minimize, or avoid complications of diabetes.

### Measurement

The following measurement would be taken during the data collection they are as follows:

1. Sociodemographic variables: The sociodemographic variables of the participants will be recorded before the commencement of the study. These include age, race, gender, height, weight, educational level, duration of diabetes, level of pain, blood pressure, pulse rate, and heart rate.
2. Pain intensity: The alternate visual analog scale will be used to assess the pain level of the participants. The participants will be requested to rate on a 0-10 scale, the level of pain they felt 6 weeks before and at present. On the scale, 0 indicates no pain or no interference, whereas 10 is pain as bad as it could be/extreme interference [26]. The alternate visual analogue scale has been reported to have a high validity, increased patient compliance, greater sensitivity of measurement, and reduced bias [26].
3. BDI: This will be used to assess and screen participants for depression. The BDI is a 21-item multiple choice self-reported inventory, widely used to assess the presence and degree of depression in adolescents and adults [27]. The BDI is a self-administered questionnaire and it has a high test-retest reliability (Pearson *r*).
4. QoL: The General Health Survey Short Form (SF-36) will be used to measure changes in the participant’s QoL. The instrument has shown good validity and reliability of Cronbach alpha of .85 and reliability coefficient of .75 for all the domains except social functioning [28]. The short form, SF-36, measures perceived health in the areas of physical functioning, role-physical, bodily pain, general health, vitality, social functioning, role-emotional, and mental health, with higher scores (range 0-100) reflecting better-perceived health.
5. Biochemical parameters: Fasting blood glucose in mmol/L, blood total cholesterol (TC) in mmol/L, low-density lipoprotein (LDL) mmol/L, very low-density lipoproteins (VLDL) mmol/L, and high-density lipoprotein (HDL) mmol/L will be determined at baseline and after 8 weeks of the program. A blood sample will be taken by pinprick using Accu-check Sofclix Pro Roche Diabetes care, South Africa. Then, the following parameters will be evaluated: fasting glucose level using Accutrend (Roche Diagnostics, GmbH Germany); HDL-C using PEG þ cholesterol oxidase (Roche Diagnostics GmbH Germany); triglycerides using glycerol phosphate-PAP (Roche Diagnostics GmbH Germany); and TC using cholesterol oxidase-PAP (Roche Diagnostics GmbH Germany) to evaluate TC, LDL, VLDL, and HDL-C.
6. Pulmonary parameters: Pulmonary function parameters will be measured at baseline and after the 8 weeks of the study using Spiro 232 (PK Morgan, India). The following parameters will be measured: Forced expiratory volume in first second (FEV_1_) (It), forced vital capacity (FVC), FEV_1_/FVC ratio, peak expiratory flow rate, and maximal voluntary ventilation (It/min). The spirometer will be connected to a computer that will record all the values.

### Sample Size and Power Calculation

The sample size (N) will be determined using Cohen table [29] at alpha=.05 and degree of freedom (µ)=k−1, where k is the number of groups, with effect size f=0.35. From the previous study [17] and power (w) 80%, sample size n=20. Sample size (N) will be 60, and 12 extra participants will be added to make room for attrition. Therefore, the sample size will be 72 participants, and each group will be 24 participants.

### Randomization and Blinding

**Figure 1:**
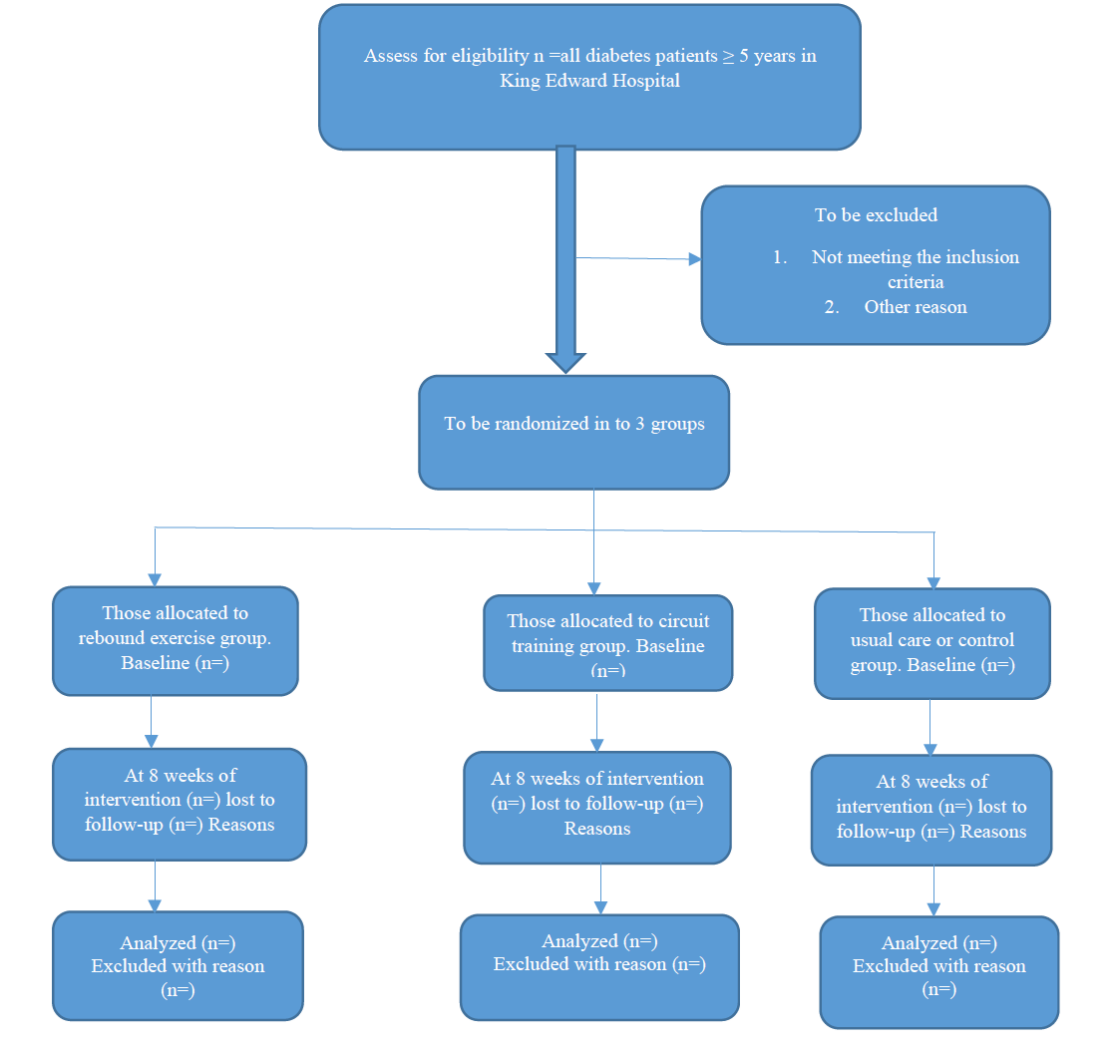
CONSORT flow chart.

Randomization will be conducted using a computer-generated random allocation sequence schedule conducted by a physiotherapist who is not involved in the research and has experience in research, who will randomly allocate recruited participants into the rebound exercise group, circuit training group, and the control group. To eliminate bias, the assessment of outcome will be performed by experienced assessors who will be blinded to the type of intervention as well as the intervention groups of the participants. Participants will also be instructed not to disclose their individual intervention groups to the assessors. Figure 1 shows the flow diagram.

### Procedures

Assessment of participants will be conducted at 2 stages: at baseline and at the end of 8 weeks of intervention. To ensure a comprehensive assessment, a battery of measures was chosen covering the WHO-ICF model [30]. Certain tools were selected to cover the 3 key domains proposed by the ICF: body structures and function (hematological variables, MSKP, and respiratory parameters), activity, and participation (QoL and depression). At baseline, participants will be assessed for sociodemographic characteristics, which will include personal demographic information and diabetes-specific information. The personal demographic information will include age, sex, height, weight, marital status, educational qualification, and employment. The diabetes-specific information will include type of diabetes and duration since diagnosis. At the baseline, the outcome measures to be used for these assessments are presented in the measurement section. Table 1 shows the instruments.

### Data Management

Data from the trial will be monitored by regularly scrutinizing data files for omissions and errors. All manually entered data will be double entered, and the source of any inconsistencies will be explored and resolved. Electronic data will be stored on password-protected servers at the Department of Physiotherapy, School of Health Sciences, University of KwaZulu-Natal, Durban, and written forms and data will be stored in locked filing cabinets at the Department of Physiotherapy, School of Health Sciences, University of KwaZulu-Natal, Durban. Data will only be accessible to the researcher. Each participant in the study will be provided with an identification number. All recorded data will be coded using this number.

**Table 1:** Study assessment tools.

Scales or tools	Function or applications
Metronome	To provide signals for participants during exercises
Mini trampoline	For rebound exercise
Treadmill	For aerobic component of circuit training
Biodata form	To record the sociodemographic variables of participants
Alternate visual analog scale	To assess change in pain scores of participants
Beck Depression Inventory	To assess change in depression of participants
General health survey short form (SF-36)	To assess change in quality of life of participants
Accutrend plus	To assess the fasting glucose-level change and cholesterol-level change in participants
Spiro232	The spirometer will be used to measure change in forced expiratory volume, forced vital capacity, peak expiratory flow rate, and maximal voluntary ventilation

### Data Analysis

Preliminary analysis will be performed to test the normality of the data using the Kolmogorov V-Smirnov test before the analysis. Demographic data will be analyzed using descriptive statistics, whereas data on QoL and depression will be analyzed within the group using the Wilcoxon signed rank test and between the groups using the Kruskal-Wallis test. Post hoc test will be used for any significant F values.

The paired *t* test will be used within the group for pain scores, fasting glucose, and cholesterol level of the participants and between groups using 1-way multivariate analysis of variance. Post hoc test will be used for any significant F values. The level of significance will be set at .05. The SPSS version 23 will be used for the analysis.

### Ethics and Informed Consent

Ethical approval was sought and obtained from the University of KwaZulu-Natal Biomedical Research Ethics Committee South Africa (BREC/BF/371/17) and registered with Clinicaltrials.gov: NCT03200795. Informed consent will also be obtained from the participants.

## Results

The results of this study are expected to provide additional information on the effect of rebound exercise and circuit training in the management of patients with musculoskeletal complications of diabetes in South Africa. It is hoped that it will lead to improvement in the QoL, reduction in MSKP and depression, improvement in glycemic control, reduction in cholesterol level, as well as improvement in respiratory capacity of type 2 diabetes patients. It is also expected to give information about which of the exercises is most effective in the management of patients with musculoskeletal diabetes complications in terms of pain relief, glycemic control, cholesterol level, improvement in QoL, level of depression, and respiratory capacity.

## Discussion

Though different additional strategies such as exercise and dietary and lifestyle modifications exist for the control of type 2 diabetes, they are mostly applied for the control of glucose level. No strategies have been identified for the control of complications associated with diabetes such as MSKP, depression, and reduction in the QoL. To date no additional rehabilitation [31] that is aimed at alleviating MSKP associated with diabetes complication. Exercises in the management of type 2 DM are of different type of different modes and intensity, most of the exercises are aerobic in nature and involves weight bearing, and strength training, mostly targeted for glycemic control, cholesterol, and few in QoL.

Exercises that are nonweight bearing, such as rebound exercises, and that require less equipment are used in the management of type 2 diabetes [23]. Studies on rebound exercises mostly focus on the glycemic control and change in cholesterol level. This trial is focused on the effect of exercises such as rebound exercise on respiratory parameters, MSKP, and QoL. The current literature suggests that emphasis in any current trial in the management of type 2 DM should focus on QoL, respiratory capacity, and psychosocial variables [32-34]. Moreover, if studies on exercises among patients with diabetes focus on respiratory and musculoskeletal complications, they are likely to result in improved functions and reduce complications.

The possible limitation of this protocol is that it is limited to the short-term effect. It is hoped that subsequent trials will look into the long-term effect of both rebound exercise and circuit training in the management of MSKP associated with diabetes.

## Abbreviations

BDI: Beck Depression Inventory
DM: diabetes mellitus
FEV: forced expiratory volume
FVC: forced vital capacity
HDL: high-density lipoprotein
ICF: International Classification of Functioning Disability and Health
IDF: International Diabetes Federation
LDL: low-density lipoprotein
MSKP: musculoskeletal pain
6MWT: 6 min walk test
QoL: quality of life
RCT: randomized controlled trial
SF-36: short form 36
VLDL: very low-density lipoprotein
